# Draft Genome Sequence of *Bacillus thuringiensis* INTA Fr7-4

**DOI:** 10.1128/genomeA.00076-17

**Published:** 2017-03-30

**Authors:** Laura E. Navas, Marcelo F. Berretta, Elio M. Ortiz, Diego H. Sauka, Graciela B. Benintende, Rubén O. Zandomeni, Ariel F. Amadio

**Affiliations:** a Instituto de Microbiología y Zoología Agrícola, Instituto Nacional de Tecnología Agropecuaria (INTA), Castelar, Buenos Aires, Argentina; b Estación Experimental Agropecuaria Rafaela, Instituto Nacional de Tecnología Agropecuaria (INTA), Rafaela, Santa Fe, Argentina; c Consejo Nacional de Investigaciones Cientíﬁcas y Técnicas (CONICET), Buenos Aires, Argentina

## Abstract

We report here the complete annotated 6,035,547-bp draft genome sequence of *Bacillus thuringiensis* INTA Fr7-4. This strain contains three *cry8* and two *vip1* and *vip2* insecticidal toxin genes.

## GENOME ANNOUNCEMENT

*Bacillus thuringiensis* is a ubiquitous Gram-positive spore-forming bacterium which produces parasporal inclusions (crystals) during sporulation composed of proteins toxic to different insects. In recent years, the number of genome sequences of *B. thuringiensis* has increased as an attempt to discover new insecticidal proteins useful for biocontrol of agricultural pests and mosquitoes. *B. thuringiensis* INTA Fr7-4 is a strain isolated from a soil sample in the province of Misiones, Argentina. We have previously reported the complete sequences of four plasmids from this strain named pFR12, pFR12.5, pFR55, and pFR260, according to their length in kilobase pairs (1, 2). We have also characterized the insecticidal genes *cry8Kb3*, *cry8Pa3,* and *cry8Qa2* (3, 4) present in a pathogenicity island, along with two vip2-vip1 operons in pFR260 (2).

In this study, genomic DNA from *B. thuringiensis* INTA Fr7-4 was used to construct a paired-end library using long-jumping-distance technology, with an insert size of 8 kbp. It was sequenced by a 2 × 150-bp run on an Illumina MiSeq (MWG Euroﬁns), generating 4,884,828 paired-end reads with an average length of 129 bp, and 4,962,965 singleton reads averaging 124 bp in length.

A *de novo* assembly was done using Velvet (5). As a result, 7,014,713 reads were assembled in 154 contigs and 12 scaffolds longer than 6 kbp. The longest scaffold resulted in 3.9 Mbp. *In silico* gap filling was performed with GapFiller 1.10 (6), closing 12 gaps and adding 13,149 bp to the scaffolds. The ﬁnal assembly of *B. thuringiensis* INTA Fr7-4 presented a total size of 6,035,547 bp.

The 12 scaffolds were compared to the GenBank nonredundant database using BLASTN. Five of them (totaling 5,233,368 bp) cover 98% of the chromosome of *Bacillus thuringiensis* serovar Indiana strain HD521 (7) and that of a closely related strain previously classified as *Bacillus bombysepticus* strain Wang (8), both with 99% identity. The average G+C content of the chromosomal scaffolds from INTA Fr7-4 strain is 35.19%. Another five scaffolds represent plasmids pFR55 and pFR260. The two remaining scaffolds showed similarity to reported plasmids of the genus. Plasmids pFR12 and pFR12.5 were not represented in the 12 scaffolds obtained, probably due to their loss during genomic DNA extraction or library construction. The average G+C content of plasmid scaffolds is 32.71%.

Genome annotation was added by the NCBI Prokaryotic Genome Annotation Pipeline (http://www.ncbi.nlm.nih.gov/genomes/static/Pipeline.html) and the RAST server (9). tRNA and rRNA genes were identiﬁed by tRNAscan-SE (10) and RNAmmer (11), respectively. Annotation by RAST predicted 6,092 coding sequences. The whole genome contains 86 tRNA genes and seven copies of 23S/5S and 16S rRNA genes. Both the tRNA and rRNA genes are located in the chromosomal scaffolds. The BtToxin_Scanner tool (12) was used to find new insecticidal toxin genes present in the *B. thuringiensis* INTA Fr7-4 genome, but only the reported *cry8* and *vip* genes present in pFR260 were detected.

### Accession number(s).

This whole-genome shotgun project has been deposited in DDBJ/ENA/GenBank under the accession no. MSFC00000000. The version described in this paper is version MSFC01000000.
